# Shifts in the Holstein dairy cow milk fat globule membrane proteome that occur during the first week of lactation are affected by parity

**DOI:** 10.1186/s40104-020-00478-7

**Published:** 2020-07-17

**Authors:** Mallory C. Honan, Megan J. Fahey, Amanda J. Fischer-Tlustos, Michael A. Steele, Sabrina L. Greenwood

**Affiliations:** 1 Department of Animal and Veterinary Sciences, The University of Vermont, Burlington, VT 05405 USA; 2 Department of Animal Biosciences, University of Guelph, Guelph, ON N1G 2W1 Canada; 3 Department of Agriculture, Food & Nutritional Science, University of Alberta, Edmonton, AB T6G 2P5 Canada

**Keywords:** Colostrum, LC-MS/MS, Parity

## Abstract

**Background:**

The milk fat globule membrane (MFGM) proteomes of colostrum and transition milk are rich sources of proteins that are likely important for neonatal calf health. In addition, characterization of these proteomes could also yield valuable information regarding mammary gland physiology of the early postpartum lactating cow. The objectives of this research were to characterize the MFGM proteomes of colostrum and transition milk through sample collections at four timepoints postpartum, including the first milking (M1, colostrum), second milking (M2, transition milk), fourth milking (M4, transition milk), and fourteenth milking (M14, mature milk), and compare these proteomes between multiparous (MP; *n* = 10) and primiparous (PP; *n* = 10) Holstein dairy cows. Isolated MFGM proteins were labeled using Tandem Mass tagging and analyzed using liquid chromatography-tandem mass spectrometry (LC-MS/MS). Protein identification was completed using MASCOT and Sequest in Proteome Discoverer 2.2. The scaled abundance values were analyzed using PROC MIXED in SAS to determine the effects of milking (MIL), parity (PAR), and MIL × PAR. The adaptive false-discovery rate (FDR)-adjusted *P* values were determined using PROC MULTTEST. Protein characterization and bioinformatic analysis were completed using a combination of PANTHER, Blast, and Uniprot.

**Results:**

A total of 104 common proteins were identified in each of the MFGM samples. Statistical analysis revealed that 70.2% of identified proteins were affected by MIL. Of these, 78.1% were lower in M14 compared with M1, including immune-related proteins lactotransferrin, lactadherin and hemopexin. Parity affected 44.2% of proteins. Of the proteins affected by PAR, 84.8% were higher in MP cows compared with PP cows, including apolipoprotein E and histones 2A, 2B, 3, and 4 b. Butyrophilin subfamily 1 member 1A and annexin 5 were higher in samples from PP cows. Milking × parity affected 32.7% of identified proteins, including lactotransferrin, gelsolin, vitamin D binding protein, and S100 proteins.

**Conclusions:**

This research supports previous findings that the Holstein MFGM proteome changes rapidly during the first week of lactation. In addition, this research identifies the impact of parity on the colostrum and transition milk MFGM proteomes, which may be important for milk-fed calf health or for the identification of protein biomarkers for mammary functionality.

## Background

The bovine milk fat globule membrane (MFGM) proteome constitutes 1–4% of milk protein [1] and approximately 22% of the milk fat droplet [2]. Butyrophilin subfamily 1 member 1A is highly abundant in the MFGM and comprises up to 40% of its proteome [3, 4]; however, the MFGM contains a diverse protein profile of several hundred proteins [5–7]. Biologically, the inclusion of intact MFGM in the diet supports the positive establishment of a healthy gut microbiome and protects against inflammation [8, 9]. Many of the proteins consistently identified within the MFGM support host immunity [10, 11] and also provide protection against enzymatic digestion [12]. Immune-associated bioactive properties of the MFGM may be of particular relevance in calf feeding protocols that include colostrum, transition milk, and mature milk [13–16].

The whey proteome rapidly shifts during the colostrum and transition milk period [5, 17–19], and examination of the colostral whey proteome’s bioactivity [15] and sensitivity to processing [18, 20] has been explored. While some research has also explored the exosomal [21] and MFGM [5, 22] proteomes during this period, the profile of the MFGM-associated proteome during this early postpartum period is relatively poorly characterized. Reinhardt et al. [5] identified a higher abundance of proteins related to lipid transport synthesis in the MFGM of milk collected 7 d postpartum compared with the colostrum MFGM. Additionally, bioactive proteins such as xanthine dehydrogenase, butyrophilin, and adipophilin (also known as perilipin 2) were also higher in abundance in the transition milk MFGM compared with the colostrum MFGM [5].

Apart from being important nutritionally for the calf, characterization of the MFGM may also be valuable in developing our understanding of lactation physiology because the MFGM appears to be reflective of mammary secretory cell activity [3]. Identification and use of MFGM proteins as biomarkers of mammary health and functionality could enhance our diagnostic capabilities in the field to identify cows with mammary dysfunction or estimate relative mammary performance of individual cows.

The impact of parity on milk yield and milk profile, particularly fat content [23, 24], has been well documented. Due to allometric mammary tissue growth, primiparous (PP) heifers typically have higher energetic requirements during their first cycle of lactogenesis as compared to mature multiparous (MP) cows [25–27]. This additional mammary-driven energetic requirement may result in differential profiles of the MFGM proteome from cows of different maturities, as secretory mechanisms have reliance on cellular metabolism. For this research, it was hypothesized that the MFGM proteome will shift during the early postpartum period, specifically during the transition from colostrum to mature milk production. It was further hypothesized that parity would differentially affect this response due to the continued mammary gland development in PP versus MP cows. The objectives of this research were to characterize dynamic shifts in the MFGM proteome of both PP and MP Holstein cows across the first, second, fourth and fourteenth milking postpartum and comparatively analyze the impact of parity on this proteome.

## Materials and methods

Experimental procedures were conducted in accordance with the Canadian Council of Animal Care [28] and all procedures were approved by the University of Alberta Animal Care and Use Committee for Livestock (AUP 00002015).

### Animals and sample collection

Twenty Holstein dairy cows that were group-housed at Breevliet Farms Ltd. (Alberta, Canada) were included in the study. As outlined by Fischer-Tlustos et al. [29], all cows were fed the same dry cow diet before parturition and the same lactating cow diet after parturition. No cows displayed clinical signs of illness during the sampling period. For this trial, milk samples were collected from 10 PP cows and 10 MP cows (parity = 3.1 ± 0.43). As described by Fahey et al. [30], all cows were milked twice daily (05:00 h and 16:00 h) and milk samples were collected from both groups using continuous in-line samplers at four milkings after parturition: 1) at the first milking postpartum (M1: 5.3 ± 0.73 h after parturition; colostrum), 2) the second milking postpartum (M2; transition milk), 3) the fourth milking postpartum (M4; transition milk), and 4) the fourteenth milking postpartum (M14; mature milk). Milk yield and fat, protein, lactose, total solids, milk urea nitrogen, somatic cell count, and immunoglobulin G concentrations across milkings are outlined by Fischer-Tlustos et al. [29]. Aliquots of milk samples for the experiment presented herein were collected into 15 mL non-sterilized tubes (Catalogue# 14-959-53A, Thermo Scientific, Rockford, IL, USA), snap frozen in a dry ice/ethanol bath immediately after collection as per methods by Tacoma et al. [18], transported on dry ice to the University of Alberta (Edmonton, AB, Canada), and stored at − 80 °C. Samples were then shipped on dry ice to the University of Vermont (Burlington, VT, USA), and stored at − 80 °C until proteomic analysis.

### MFGM protein fractionation

Samples were thawed overnight at 4 °C, and 400 μL of protease inhibitor cocktail (Protease Inhibitor Cocktail, Catalogue # P8340, Sigma Aldrich, St. Louis, MO, USA) was added to each tube. Two samples were deemed unusable during this preliminary processing due to transport damage, hence the remaining 78 samples were further processed. These 78 milk samples were centrifuged at 4,000×*g* at 4 °C and the cream layer was collected using a clean spatula and placed into a new 15-mL tube. This separation step, including centrifugation and separation of the cream layer, was repeated. The cream layer was stored at − 80 °C for MFGM proteome analysis.

Sample processing was performed as per methods established by Yang et al. [1] with minor modifications described herein. For proteomic analysis, up to 10 volumes of phosphate buffered saline (PBS) was pipetted into each thawed sample and vortexed. All samples were then incubated for 20 min at 37 °C, centrifuged at 4,000×*g* for 30 min, and PBS was aspirated. The addition of PBS, followed by a 20 min incubation at 37 °C, centrifugation at 4,000×*g* for 30 min, and aspiration of the PBS, was repeated twice more for a total of three washes.

After washing with PBS, the cream was transferred into a new 50-mL round-bottom Nalgene tube (Catalogue#79013, United States Plastic Corp., Lima, OH, USA). Five volumes of lysis buffer (50 mmol/L Tris-HCl at pH 7.4, 4% SDS (wt/vol) solution) was added to each tube and vortexed. These samples were incubated at room temperature for 1 h with periodic vortexing every 10–15 min and then subsequently incubated at 95 °C for 5 min. Samples were then centrifuged at 12,000×*g* for 15 min and the resulting fat layer was removed. The samples were again centrifuged at 12,000×*g* for 15 min and any residual fat was removed. The aqueous phase was collected through a transfer pipette and deposited into a new 15-mL tube. An aliquot was then combined with acetone at a 1:6 ratio (sample: acetone) and incubated at − 20 °C for 20 h immediately after mixing. Samples were then centrifuged at 14,000×*g* for 20 min at 4 °C and the subsequent supernatant was discarded. Radioimmunoprecipitation assay (RIPA) buffer (Thermo Scientific, Rockford, IL, USA) was used to resuspend the pellet before storage at − 80 °C.

### Protein quantification and isobaric TMT labeling

Processed samples were thawed on ice. To create one universal control (UC) that could later be used to compare against each individual sample, a composite UC mixture was created by combining aliquots of each animal. The final volume of UC was enough to later generate 9 identical aliquots from this one composite mixture for inclusion in each multiplex submitted for LC-MS/MS analysis. The protein concentration of each individual sample (*n* = 78), as well as the UC (*n* = 1), was then determined using a bicinchoninic assay (BCA; Catalogue #23225, Pierce Biotechnology, Rockford, IL, USA) kit. Samples and the UC were then subjected to isobaric labeling using Tandem Mass Tag™ (TMT™) 10plex Isobaric Labeling Kits (Pierce Biotechnology, Rockford, IL, USA). An aliquot of the UC was included as one sample in every multiplex to ensure consistent labeling and loading. In total, 9 multiplexes were created, each containing the UC and a randomized subset of the samples in order to complete a comparative analysis of the 78 samples. All LC-MS/MS analysis was completed at The Vermont Genetics Network Core Proteomics Facility (Burlington, VT, USA).

### Liquid chromatography-tandem mass spectrometry (LC-MS/MS)

The purified TMT-labeled and combined peptides were resuspended in 2.5% acetonitrile (CH_3_CN) and 2.5% formic acid (FA) in water for subsequent LC-MS/MS based peptide identification and quantification. Analyses were performed on the Q-Exactive mass spectrometer coupled to an EASY-nLC ULTRA (Thermo Scientific, Waltham, MA, USA). Samples were loaded onto a 100 μm × 500 mm capillary column packed with Halo C18 (2.7 μm particle size, 90 nm pore size, Michrom Bioresources, CA, USA) at a flow rate of 300 nL/min. Program settings and parameters for LC-MS/MS analysis were performed as outlined by Scuderi et al. [31]. Briefly, the column end was laser pulled to a ~ 3 μm orifice and packed with minimal amounts of 5um Magic C18AQ before packing with the 3-μm particle size chromatographic materials. To separate peptides, the following gradient was used: 2.5–35% CH_3_CN/0.1% FA over 150 min, 35–100% CH_3_CN/0.1% FA in 1 min and then 100% CH_3_CN/0.1% FA for 8 min, followed by an immediate return to 2.5% CH_3_CN/0.1% FA and a hold at 2.5% CH_3_CN/0.1% FA. A nanospray ionization source with a spray voltage of 2.0 kV was used to introduce peptides. Mass spectrometry data was acquired in a data-dependent “Top 10” acquisition mode with lock mass function activated (*m/z* 371.1012; use lock masses: best; lock mass injection: full MS). A survey scan from *m/z* 350–1600 at 70,000 resolution (AGC target 1e^6^; max IT 100 ms; profile mode) was followed by 10 higher-energy collisional dissociation (HCD) tandem mass spectrometry (MS/MS) scans on the most abundant ions at 35,000 resolution (AGC target 1e^5^; max IT 100 ms; profile mode). MS/MS scans were acquired with an isolation width of 1.2 *m/z* and a normalized collisional energy of 35%. Dynamic exclusion was enabled (peptide match: preferred; exclude isotopes: on; underfill ratio: 1%). Protein identification was completed using MASCOT and Sequest in Proteome Discoverer 2.2 (Thermo Scientific, Waltham, MA, USA) against a curated Uniprot *Bos taurus* protein database (3AUP000009136). The raw files were searched against the database as one contiguous input file, resulting in one result file. The peptide abundances in the labeled UC samples were set as 100 and the abundances of the proteins in the 78 experimental samples were scaled accordingly.

### Statistical and bioinformatic analysis

The scaled abundance values of the identified proteins were statistically analyzed. Statistical analysis was performed using PROC MIXED of SAS (Version 9.4) and included cow as the subject and milking (MIL) as the repeated measure. The effects of MIL, parity (PAR), and MIL **×** PAR were determined. The adaptive false-discovery rate (FDR) adjusted treatment effects [32] were determined using PROC MULTTEST to account for multiple hypotheses testing. The accession number of the proteins that were labeled as “uncharacterized” were searched against the UniProt [33] database to obtain their FASTA sequences. The FASTA sequences of these proteins were then searched against the PANTHER version 14.1 [34] or BLAST [35] databases to identify the protein name. Accession numbers of identified proteins were searched against the the PANTHER *Bos taurus* database [34] to ascertain gene ontology (GO) annotations of identified proteins. Proteins were classified according to their biological processes. All GO classifications presented herein are presented as the percent of gene hits against the total number of genes.

## Results

### Protein profile of the identified MFGM proteome

**Fig. 1:**
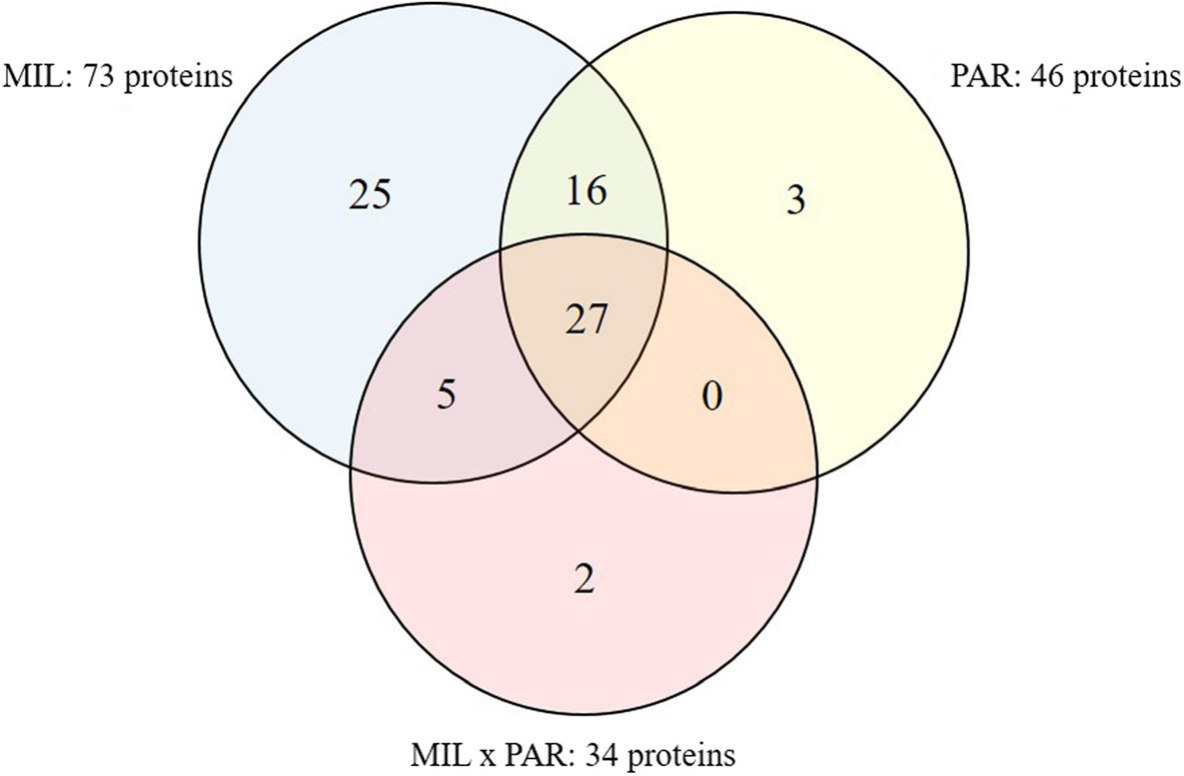
Number of proteins affected by milking (MIL), parity (PAR), and MIL × PAR within the milk fat globule membrane (MFGM) collected from 10 primiparous (PP) and 10 multiparous (MP) Holstein dairy cows at four milkings postpartum (M1, M2, M4, M14)

**Fig. 2:**
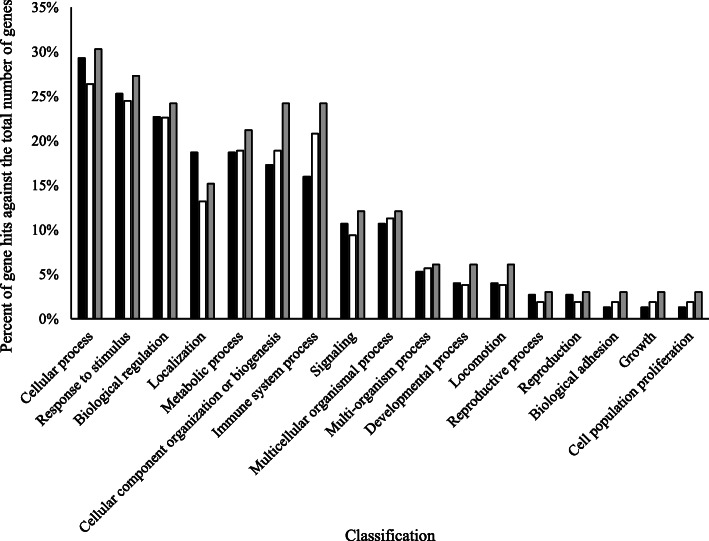
Biological processes of the total proteome identified in the current trial (black bars), the proteins affected by milking (white bars), and the proteins affected by parity (grey bars)

Of the 104 proteins identified and analyzed in this study, the abundance of 78 proteins (75%) changed in response to MIL, PAR, and/or MIL × PAR (Fig. 1). Xanthine dehydrogenase/oxidase, serum albumin, lactadherin, lactotransferrin, butyrophilin subfamily 1 member A1, and perilipin were present at the highest peptide counts (Supplementary Table S1). Annotation of the total proteome included cellular process (29%), response to stimulus (25%), and biological regulation (23%) as the three primary biological process classifications encompassed by this proteome (Fig. 2). Differences between the total proteome GO profile and GO profiles of proteins affected by MIL and PAR were evident (Fig. 2): PAR impacted a greater proportion of proteins associated with cellular component organization and immune system processes, and MIL and PAR affected fewer proteins involved in localization compared with their representation in the total proteome.

### Proteins affected by MIL

**Fig. 3:**
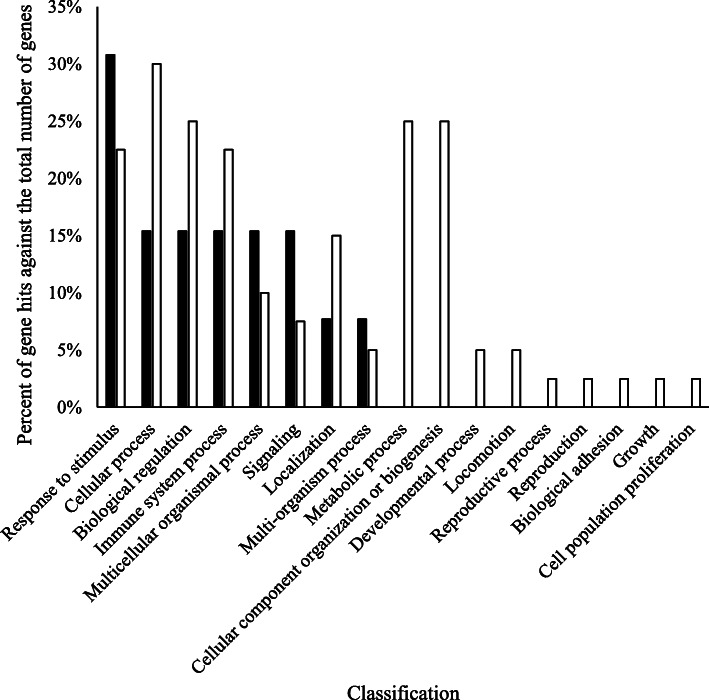
Biological processes of proteins that were impacted by milking. Proteins that were higher at the fourteenth milking postpartum (M14) compared with the first milking postpartum (M1) are displayed in black. Proteins that were lower at the fourteenth milking postpartum (M14) compared with the first milking postpartum (M1) are displayed in white

Milking affected 73 (70.2%) of the 104 identified proteins. Of the 73 proteins impacted by MIL, 57 (78.1%) were lower in abundance in M14 samples compared with M1 samples (Table 1). Examples of proteins within this grouping were lactotransferrin, lactadherin, vitamin D binding protein, hemopexin, and several immune-associated proteins (examples: IgA, IgJ, IgK, IgL, IgM, CD177, CD5, serum amyloid A). Conversely, 16 proteins (21.9%) were higher in abundance in M14 samples compared with M1 samples (Table 2). Proteins in this grouping included butyrophilin subfamily 1 member 1A, perilipin, and several binding proteins. Dominant GO classifications of these protein groupings is comparatively represented in Fig. 3. Higher representation of proteins involved in cellular process, biological regulation, immune system process, and localization was observed in proteins that were lower in abundance in M14 compared with M1. Proteins involved in the response to stimulus, multicellular organismal process, and signaling were more represented in the group of proteins of higher abundance in M14 compared with M1.

**Table 1:** Milk fat globule membrane proteins secreted by 10 primiparous (PP) and 10 multiparous (MP) Holsteins at four milkings postpartum (M1, M2, M4, M14) that were affected by milking (MIL) and had lower abundance at M14 compared with M1

Accession number	Description	M1		M2		M4		M14		*P*-value
		Value	SE	Value	SE	Value	SE	Value	SE	
A0A0A0MP90	Histone H2A	92.0	26.1	106.2	15.5	103.2	24.2	30.7	6.2	<.0001
A0A0N4STN1	Cathelicidin-1-like	116.1	14.6	100.6	10.6	96.2	7.3	63.7	8.6	0.0043
A0A140T881	Apolipoprotein E	116.8	11.5	110.7	10.1	100.8	5.7	78.5	6.7	0.0079
A8DC37	Fc-gamma-RII-D	154.7	14.3	148.0	17.8	84.6	7.5	44.8	3.2	<.0001
E1BF48	CD177 molecule	94.8	27.0	108.1	14.7	97.0	18.8	32.8	5.9	<.0001
E1BGN3	Histone H3	83.1	27.1	97.1	12.7	102.3	22.6	36.6	6.1	<.0001
F1MCF8	IGL@ protein	146.8	34.6	86.4	9.8	56.0	7.4	21.1	2.7	<.0001
F1MH40	IGK protein	151.6	26.9	99.3	9.2	64.7	7.8	29.4	3.1	<.0001
F1MHH9	Low affinity immunoglobulin gamma Fc region receptor II	146.2	22.9	112.7	15.9	80.8	9.5	45.1	3.8	<.0001
F1MHS5	Protein S100-A9	115.2	26.6	109.7	16.2	99.8	16.3	32.0	5.3	<.0001
F1MLW8	Immunoglobulin lambda-1 light chain-like isoform X5	133.9	20.2	84.2	10.4	69.3	16.6	35.2	3.7	<.0001
F1MLZ1	Cytochrome b reductase 1	143.2	25.3	95.9	16.0	77.8	8.5	39.3	5.0	<.0001
F1MMW8	Serum amyloid A protein	116.1	11.0	118.7	18.7	108.5	10.4	69.4	8.1	0.0016
F1MUD2	Histone H2B	88.5	36.8	102.2	14.1	104.3	26.5	32.8	6.7	<.0001
F1MX83	Protein S100	134.6	19.0	119.9	9.4	95.4	8.5	61.6	6.3	<.0001
F1MXX6	Lactadherin	130.5	11.0	118.7	12.8	92.9	8.4	75.6	8.9	0.001
F1N1I6	Gelsolin	124.6	13.1	107.7	9.1	95.2	6.1	65.5	5.9	<.0001
F1N4Y5	Non-classical MHC class I antigen precursor	131.4	10.1	130.4	15.1	94.8	7.8	68.4	5.4	<.0001
F1N514	CD5 antigen-like precursor	153.3	14.0	115.8	12.5	62.4	4.8	32.7	3.1	<.0001
F1N5M2	Vitamin D-binding protein	147.1	17.2	96.6	11.2	67.9	6.5	40.0	3.4	<.0001
F1N650	Annexin	97.5	29.5	86.1	14.9	117.3	19.2	50.5	7.6	0.0044
F1N726	Glycoprotein 2	97.9	12.3	132.2	13.7	94.6	11.7	51.1	7.7	<.0001
G3MWV5	Histone cluster 1 H1 family member e	89.9	41.4	94.2	13.7	86.9	23.5	21.9	3.3	<.0001
G3MXB5	Immunoglobulin IgA heavy chain constant region, partial	110.4	13.3	132.8	13.8	69.7	8.4	26.0	4.7	<.0001
G3N0V0	Secreted immunoglobulin gamma2 heavy chain constant region, partial	137.1	37.2	67.9	10.2	81.0	26.7	30.1	5.3	0.0005
G3N2D7	Immunoglobulin light chain, partial	105.0	9.0	83.0	6.8	76.1	6.5	45.8	4.2	<.0001
G3X7A5	Complement C3	123.4	17.5	91.1	8.8	102.2	12.6	71.1	6.5	0.0124
G5E513	IgM heavy chain constant region, secretory form, partial	150.9	14.4	119.0	14.7	65.2	5.2	36.5	3.3	<.0001
G5E5T5	Immunoglobulin M heavy chain secretory form	146.2	13.8	117.2	13.2	67.6	5.1	45.8	3.7	<.0001
G5E5V1	Immunoglobulin iota chain-like, partial (TPA)	135.3	21.0	94.0	8.0	65.1	8.9	28.9	4.9	<.0001
G5E604	TPA: immunoglobulin iota chain-like	136.0	16.6	95.9	12.3	66.4	12.1	34.6	4.1	<.0001
P01888	Beta-2-microglobulin	110.0	46.5	93.0	16.6	90.1	16.4	35.3	5.6	0.0004
P08037	Beta-1,4-galactosyltransferase 1	127.3	13.3	132.0	17.3	92.3	13.9	54.1	5.9	<.0001
P08728	Keratin, type I cytoskeletal 19	116.9	10.5	134.6	13.1	91.0	8.2	51.4	4.6	<.0001
P13753	BOLA class I histocompatibility antigen, alpha chain BL3–7	107.6	10.0	127.8	22.4	100.8	7.8	75.1	7.0	0.0103
P15497	Apolipoprotein A-I	135.1	10.2	141.0	17.1	116.3	7.5	73.7	5.4	<.0001
P17697	Clusterin	148.6	11.2	139.7	13.8	75.3	7.4	37.1	3.8	<.0001
P19035	Apolipoprotein C-III	145.0	15.3	142.5	18.4	77.4	5.4	45.6	3.6	<.0001
P24627	Lactotransferrin	118.0	32.4	76.6	9.8	75.5	8.5	43.7	4.3	0.0003
P28782	Protein S100-A8	110.9	23.9	113.4	16.2	95.5	17.3	30.9	4.3	<.0001
P48616	Vimentin	112.6	18.7	108.2	10.5	104.8	13.4	51.7	5.5	<.0001
P56425	Cathelicidin-7	92.7	17.0	115.3	21.0	120.7	16.0	47.5	5.7	<.0001
P60712	Actin, cytoplasmic 1	126.6	19.0	109.4	10.4	90.4	7.4	58.8	5.1	<.0001
P62261	14–3-3 protein epsilon	121.9	8.6	116.9	11.2	99.3	8.1	87.8	9.4	0.0397
P62803	Histone H4	98.0	35.8	105.6	12.3	108.2	23.7	36.4	6.4	<.0001
P62992	Ubiquitin-40S ribosomal protein S27a	129.0	11.2	112.8	11.3	113.8	11.1	90.2	7.8	0.0338
P79105	Protein S100-A12	116.4	16.1	121.4	14.7	93.3	17.4	30.3	5.0	<.0001
P81265	Polymeric immunoglobulin receptor	112.9	12.4	122.5	13.5	80.9	6.6	40.6	4.8	<.0001
P81644	Apolipoprotein A-II	120.1	13.0	128.3	14.9	102.2	7.1	85.2	8.8	0.041
Q0VCN9	Folate receptor 2 (Fetal)	150.4	11.0	121.9	13.7	78.1	4.3	43.3	3.1	<.0001
Q17QG8	Histone H2A	81.5	27.4	105.7	15.1	96.6	21.2	29.6	5.7	<.0001
Q1JPB0	Leukocyte elastase inhibitor	132.9	23.2	93.0	10.1	86.0	8.6	46.7	6.2	<.0001
Q29S21	Keratin, type II cytoskeletal 7	111.6	9.2	131.8	12.6	102.2	7.6	74.3	5.3	<.0001
Q2KII3	Hepatitis A virus cellular receptor 1 N-terminal domain containing protein	127.3	9.2	116.9	13.3	105.8	7.6	68.3	3.9	<.0001
Q3SYR8	Immunoglobulin J chain	124.3	8.8	111.7	7.8	76.4	4.2	42.9	4.8	<.0001
Q3SZV7	Hemopexin	134.8	17.3	99.9	10.4	89.3	12.1	51.6	8.3	<.0001
Q8SQ28	Serum amyloid A protein	123.8	11.6	133.5	22.9	109.2	11.4	67.5	9.7	0.001

**Table 2:** Milk fat globule membrane proteins secreted by 10 primiparous (PP) and 10 multiparous (MP) Holsteins at four milkings postpartum (M1, M2, M4, M14) that were affected by milking (MIL) and had higher abundance at M14 compared with M1

Accession number	Description	M1		M2		M4		M14		*P*-value
		Value	SE	Value	SE	Value	SE	Value	SE	
Q8WML4	Mucin-1	63.0	4.0	80.4	6.2	154.7	16.3	220.3	29.4	<.0001
P10790	Fatty acid-binding protein, heart	68.5	5.0	78.6	7.5	144.5	14.6	235.9	32.7	<.0001
B2D1N9	ATP-binding cassette sub-family G member 2	84.4	4.1	84.0	7.4	125.0	13.1	239.3	33.6	<.0001
P80195	Glycosylation-dependent cell adhesion molecule 1	56.4	6.2	67.2	9.3	164.1	16.8	261.3	37.9	<.0001
F1N1N6	Perilipin	83.0	5.5	101.1	9.6	123.5	13.6	205.3	29.8	0.0002
P02663	Alpha-S2-casein	72.7	13.3	77.0	13.1	150.9	24.7	150.9	18.0	0.0006
P18892	Butyrophilin subfamily 1 member A1	87.3	5.0	104.3	9.3	130.3	15.4	171.0	23.7	0.0008
F1MGC2	Non-secretory ribonuclease isoform X1	87.0	8.2	92.3	13.0	150.0	21.2	140.7	13.3	0.0013
F1N6D4	Sodium-dependent phosphate transport protein 2B	95.1	5.5	96.2	9.6	126.1	12.4	175.0	23.8	0.0032
E1BHI7	Butyrophilin subfamily 1 member A1	87.9	9.4	101.2	14.2	138.9	21.8	205.4	33.7	0.0041
Q0IIG8	Ras-related protein Rab-18	98.0	4.8	103.0	9.2	126.7	11.6	161.5	21.3	0.0083
Q2KIS4	Dehydrogenase/reductase (SDR family) member 1	93.7	5.5	103.4	10.1	133.4	11.4	116.0	11.7	0.0149
P02662	Alpha-S1-casein	76.8	12.2	88.3	18.3	130.1	24.5	162.6	26.3	0.0163
P10152	Angiogenin-1	84.0	9.1	78.0	11.6	108.8	13.2	175.0	34.8	0.0298
P00711	Alpha-lactalbumin	54.8	15.0	30.7	5.1	152.9	66.0	127.6	48.8	0.0331
Q3SYS6	Calcineurin B homologous protein 1	107.0	11.5	107.0	7.5	136.2	8.4	130.8	11.4	0.0376

### Proteins affected by PAR

Parity influenced the abundance of 46 proteins and 39 (84.8%) of these proteins were higher in relative abundance in MP cows compared with PP cows (Table 3). Gelsolin, histones 1H1E, H2A, H2B, H3 and H4, apolipoprotein E, complement C3, lactotransferrin, keratin 1, and monocyte differentiation antigen CD14 are examples of proteins within this grouping. The remaining 7 proteins (15.2%) were higher in PP cows compared with MP cows (Table 4), and examples include butyrophilin subfamily 1 member A1, apoliproteins A-I and C-III, cytochrome b reductase 1, and annexin 5. Only 3 proteins were affected only by PAR and not by MIL or MIL × PAR: monocyte differentiation antigen CD14, keratin 1, and annexin 5.

**Table 3:** Milk fat globule membrane proteins that were present at lower abundance in colostrum and milk collected at milkings 1, 2, 4, and 14 postpartum from primiparous (PP; *n* = 10) cows compared with multiparous (MP, *n* = 10) cows. Average abundance across milkings is presented within parity

Accession number	Description	MP		PP		*P*-value
		Value	SE	Value	SE	
A0A0N4STN1	Cathelicidin-1-like	123.6	7.5	64.7	7.5	<.0001
F1MHS5	Protein S100-A9	126.6	12.6	51.8	12.6	<.0001
F1N726	Glycoprotein 2	128.6	8.2	59.3	8.2	<.0001
G3MXB5	Immunoglobulin IgA heavy chain constant region, partial	129.1	7.6	40.4	7.6	<.0001
P28782	Protein S100-A8	125.7	12.0	49.6	12.0	<.0001
P79105	Protein S100-A12	125.7	10.0	55.0	10.0	<.0001
P81265	Polymeric immunoglobulin receptor	123.9	7.1	54.5	7.1	<.0001
Q3SYR8	Immunoglobulin J chain	114.5	4.7	63.2	4.7	<.0001
F1MH40	IGK protein	116.7	10.5	55.8	10.5	0.0001
G3N2D7	Immunoglobulin light chain, partial	91.4	4.8	63.6	4.8	0.0001
F1MX83	Protein S100	126.6	8.4	79.1	8.4	0.0002
Q1JPB0	Leukocyte elastase inhibitor	117.1	9.7	62.2	9.7	0.0002
E1BF48	CD177 molecule	117.8	12.9	48.5	12.9	0.0003
G5E5V1	Immunoglobulin iota chain-like, partial (TPA)	104.4	8.7	57.2	8.7	0.0003
E1BGN3	Histone H3	114.7	13.4	44.9	13.4	0.0005
A0A0A0MP90	Histone H2A	118.2	13.9	47.8	13.9	0.0006
P24627	Lactotransferrin	109.2	12.4	47.7	12.4	0.0008
P56425	Cathelicidin-7	121.9	11.3	66.1	11.3	0.0008
P60712	Actin, cytoplasmic 1	117.0	8.3	75.6	8.3	0.0008
F1MUD2	Histone H2B	122.6	17.0	41.3	17.0	0.0011
Q17QG8	Histone H2A	110.6	13.5	46.1	13.6	0.0012
F1N514	CD5 antigen-like precursor	107.0	6.9	75.2	6.9	0.0018
P62803	Histone H4	123.7	16.0	50.4	16.0	0.0018
G5E513	IgM heavy chain constant region, secretory form, partial	109.7	7.6	76.1	7.6	0.0025
F1N650	Annexin	118.1	13.8	57.6	13.8	0.0027
G5E5T5	Immunoglobulin M heavy chain secretory form	109.6	7.1	78.7	7.1	0.0030
F1N5M2	Vitamin D-binding protein	103.8	7.7	72.1	7.7	0.0048
F1MCF8	IGL@ protein	104.1	13.0	51.1	13.0	0.0053
P48616	Vimentin	112.9	9.1	75.7	9.1	0.0053
G3MWV5	Histone cluster 1 H1 family member e	108.0	17.5	38.4	17.6	0.0065
F1N1I6	Gelsolin	110.8	6.4	85.6	6.4	0.0070
A0A140T881	Apolipoprotein E	113.9	6.2	89.4	6.2	0.0071
A6QNL0	Monocyte differentiation antigen CD14	125.9	7.4	97.2	7.4	0.0074
F1MLW8	Immunoglobulin lambda-1 light chain-like isoform X5	99.3	10.0	62.1	10.0	0.0108
G3N0V2	Keratin 1	110.8	5.9	88.9	5.9	0.0112
G3X7A5	Complement C3	111.1	8.6	82.7	8.6	0.0217
P01888	Beta-2-microglobulin	112.3	18.4	51.9	18.5	0.0241
G3N0V0	Secreted immunoglobulin gamma2 heavy chain constant region, partial	104.1	16.7	54.0	16.7	0.0373
G5E604	TPA: immunoglobulin iota chain-like	95.7	8.6	70.7	8.6	0.0435

**Table 4:** Milk fat globule membrane proteins that were present at higher abundance in colostrum and milk collected at milkings 1, 2, 4, and 14 postpartum from primiparous (PP; *n* = 10) cows compared with multiparous (MP, *n* = 10) cows. Average abundance across milkings is presented within parity

Accession number	Description	MP		PP		*P-*value
		Value	SE	Value	SE	
F1MLZ1	Cytochrome b reductase 1	68.3	11.1	109.8	11.1	0.0105
P15497	Apolipoprotein A-I	102.5	7.8	130.5	7.8	0.0129
F1MGC2	Non-secretory ribonuclease isoform X1	98.8	10.4	136.2	10.4	0.0130
F6QVC9	Annexin	102.0	10.4	136.3	10.4	0.0218
P02662	Alpha-S1-casein	89.8	14.9	139.1	14.9	0.0222
E1BHI7	Butyrophilin subfamily 1 member A1	108.1	15.4	158.6	15.4	0.0234
P19035	Apolipoprotein C-III	89.7	8.8	115.5	8.8	0.0410

### Proteins affected by MIL × PAR

A total of 34 proteins (32.7% of identified proteins) were influenced by the interaction of MIL × PAR, and only 43.6% of these were affected by either MIL or PAR independently (Table 5). Affected proteins include vitamin D-binding protein, lactotransferrin, complement C3, clusterin, gelsolin, and protein S100 variants. The biological processes of the proteins that had altered abundance due to the interaction of MIL × PAR were diverse and similar in profile to the biological processes of the total proteome characterized in this experiment (data not shown). Of the 34 affected proteins, 25 (73.5%) followed a similar pattern, whereby MP cows had a higher abundance compared with PP cows in M1, and by M14 the protein abundance was similar within protein across PP and MP cows. Within this grouping, protein S100 variants 8, 9, and 12 are examples of proteins displaying this pattern (Fig. 5). Of exception to this pattern were the following 9 proteins: folate receptor 2 (Fetal), apolipoprotein C-III, selenoprotein F, Fc-gamma-RII-D, BOLA class I histocompatibility antigen, alpha chain BL3–7, serum amyloid A protein, CD59 molecule (CD59 blood group), clusterin, and apolipoprotein A-I. While folate receptor 2 (Fetal), apolipoprotein C-III, Fc-gamma-RII-D, BOLA class I histocompatibility antigen, alpha chain BL3–7, serum amyloid A protein, clusterin, and apolipoprotein A-I were affected by at least 1 main effect in addition to the interaction, selenoprotein F and CD59 molecule (CD59 blood group) were impacted by MIL × PAR but were not affected by the main effects of MIL or PAR due to the pattern of change within these 2 proteins.

**Table 5:** Protein abundance within the milk fat globule membrane secreted by 10 primiparous (PP) and 10 multiparous (MP) Holsteins at four milkings postpartum (M1, M2, M4, M14) that were impacted by the interaction of milking and parity (MIL × PAR)

Accession number	Description	M1			M2			M4			M14			*P*-value
		PP	MP	SE^1^	PP	MP	SE	PP	MP	SE	PP	MP	SE	
G3MXB5	Immunoglobulin IgA heavy chain constant region, partial	61.6	159.2	18.8	68.2	197.3	19.5	16.6	122.9	11.9	15.1	37	6.7	<.0001
P81265	Polymeric immunoglobulin receptor	79.8	146	17.5	75.4	169.6	19	30.7	131.1	9.3	32.1	49.1	6.9	<.0001
Q3SYR8	Immunoglobulin J chain	89.6	159	12.4	85.5	138	11.1	40.1	112.6	6	37.4	48.4	6.7	<.0001
F1MH40	IGK protein	92.3	210.8	38.1	76.4	122.3	13	27.4	102.1	11.1	27.3	31.5	4.4	0.0001
G3N2D7	Immunoglobulin light chain, partial	89.7	120.4	12.7	71.7	94.4	9.6	43.9	108.3	9.2	49.1	42.4	5.9	0.0002
F1MCF8	IGL@ protein	92	201.6	48.9	72.4	100.5	13.9	21.4	90.6	10.5	18.6	23.6	3.8	0.0007
Q0VCN9	Folate receptor 2 (Fetal)	139.8	160.9	15.6	124.3	119.6	19.4	59	97.3	6.1	47.1	39.5	4.3	0.0007
F1N5M2	Vitamin D-binding protein	118.1	176.1	24.3	83.4	109.8	15.8	42.8	93.1	9.2	44	36	4.9	0.0008
F1MHS5	Protein S100-A9	68	162.5	37.6	69.6	149.9	23	38.6	161	23	30.8	33.2	7.5	0.0011
P24627	Lactotransferrin	54	182	45.9	53	100.2	13.8	41.2	109.8	12	42.7	44.7	6.1	0.0014
Q1JPB0	Leukocyte elastase inhibitor	85	180.7	32.8	67.2	118.8	14.3	49.2	122.8	12.2	47.3	46.1	8.7	0.0024
G5E5V1	Immunoglobulin iota chain-like, partial (TPA)	95.1	175.5	29.6	75.2	112.8	11.4	29.6	100.6	12.6	28.9	28.8	7	0.0027
P28782	Protein S100-A8	70	151.7	33.8	66.7	160.1	22.9	37	153.9	24.4	24.8	37	6	0.0031
P19035	Apolipoprotein C-III	185.1	104.9	21.7	167	117.9	26	64.7	90.1	7.6	45.2	46	5	0.0054
F1N514	CD5 antigen-like precursor	122.2	184.4	19.8	102	129.6	17.7	44.3	80.6	6.8	32.1	33.4	4.4	0.0079
A8YXY3	Selenoprotein F	119.9	117.7	11.5	133.6	105.3	13.9	95.2	125.3	6.2	119.7	101.5	11.6	0.0089
P60712	Actin, cytoplasmic 1	95.6	157.6	26.9	90	128.8	14.7	59.6	121.1	10.4	57.2	60.3	7.2	0.0105
G5E5T5	Immunoglobulin M heavy chain secretory form	117.7	174.6	19.5	104.9	129.4	18.7	47.2	87.9	7.2	45	46.6	5.2	0.0111
A8DC37	Fc-gamma-RII-D	133.6	175.8	20.3	175.1	120.9	25.8	64.1	105.1	10.7	46.8	42.8	4.6	0.0111
G5E513	IgM heavy chain constant region, secretory form, partial	122.3	179.5	20.3	104.6	133.3	20.7	43.4	86.9	7.3	34	38.9	4.7	0.0113
P79105	Protein S100-A12	81.4	151.4	22.8	82	160.8	20.7	35.3	151.3	24.7	21.4	39.2	7.1	0.0123
P13753	BOLA class I histocompatibility antigen, alpha chain BL3–7	85.2	129.9	14.1	131.4	124.2	31.6	69.6	132	11.1	77	73.2	9.8	0.0142
F1N726	Glycoprotein 2	69.7	126.1	17.4	90	174.4	19.4	38.6	150.6	16.5	39.1	63.2	10.9	0.0156
Q8SQ28	Serum amyloid A protein	146.6	101	16.4	137.4	129.5	32.4	81.2	137.2	16.1	54.4	80.7	13.7	0.0185
P01888	Beta-2-microglobulin	56.9	163.2	65.7	74.5	111.5	24.1	39.9	140.3	23.2	36.5	34.2	7.9	0.0198
Q32PA1	CD59 molecule (CD59 blood group)	134.8	109.6	9.4	117.8	107.1	15.5	85.9	130.4	13.5	100.5	123.1	20.3	0.0256
G3X7A5	Complement C3	96.4	150.4	24.8	91.3	90.8	12.5	68.2	136.2	17.7	75.1	67.1	9.2	0.0343
P17697	Clusterin	153.1	144	15.8	171.3	108.2	19.5	61	89.7	10.5	34.8	39.3	5.4	0.0357
F1MLW8	Immunoglobulin lambda-1 light chain-like isoform X5	101.6	166.3	28.6	75.1	93.3	14.7	32.9	105.8	23.5	38.7	31.7	5.2	0.0359
P15497	Apolipoprotein A-I	160.1	110.2	14.5	173.4	108.5	24.2	109.3	123.4	10.6	79.4	67.9	7.6	0.039
A0A0A0MP90	Histone H2A	58.5	125.4	37	65.4	147.1	22	42.7	163.6	34.3	24.5	36.9	8.8	0.0396
F1N1I6	Gelsolin	103.1	146	18.6	95.1	120.3	12.9	75.4	115	8.7	69	62	8.4	0.0426
A0A0N4STN1	Cathelicidin-1-like	76.7	155.5	20.6	73.3	127.8	14.9	54.9	137.6	10.3	53.9	73.4	12.1	0.0448
F1MUD2	Histone H2B	40.2	136.8	52.1	63.6	140.8	19.9	36.8	171.9	37.4	24.6	41.1	9.5	0.0463

## Discussion

In the current study, we characterized the MFGM proteomes of colostrum and transition milk and examined the impact of PAR on these proteomes. Consistent with previous reports [36], the MFGM proteome was rich in proteins involved in cellular process, regardless of MIL or PAR of the cow. Recent work by Yang et al. [37] identified the response to stimulus and localization GO classifications to encompass 19% and 18% of the colostrum proteome, respectively, which is in line with our findings. The protein profile identified in the current trial was also consistent with previous research [1, 5, 9, 37], and included the highly abundant MFGM proteins xanthine dehydrogenase/oxidase, serum albumin, butyrophilin subfamily 1 member 1A, lactadherin, lactotransferrin, and perilipin 2. The importance of these proteins in aspects of milk fat droplet formation, docking and secretion is well documented [38–40], and their higher abundance relative to other proteins is in line with other reports [1, 5, 37]. Xanthine dehydrogenase/oxidase and serum albumin were 2 of the 26 proteins that were not affected by MIL, PAR or their interaction; however, the observed shift in 78 of the identified proteins (75%) demonstrates the broad impact that mammary functionality has on the MFGM proteome.

In the research presented herein, MIL was the dominant variable affecting 70.2% of identified proteins (Tables 1 and 2). Approximately 78% of proteins affected by MIL were higher in abundance in M1 compared with M14. Metabolically, the onset of colostrogenesis signals an immense shift in protein metabolism and synthesis in the mammary gland. The high protein concentration within colostrum [29], along with increasing amino acid uptake and protein synthesis in the mammary gland [41, 42], both underscore this shift. Our observation of higher abundances of histones, actin regulators, and other indicators of protein synthesis support the inclusion of protein-mediated regulation that results in an altered MFGM proteome. While Immunoglobulin G has historically been perceived as the prime indicator of colostrum quality, other proteins may also be important contributors to the healthfulness of colostrum [18, 43]. Reinhardt et al. [5] comparatively explored the MFGM proteomes from cows at parturition and 7 d postpartum, which equates to our M1 versus M14 comparison. These researchers observed that approximately 33% of the proteome was affected by days in milk and reported that several immune-related proteins were lower at 7 d postpartum compared with the colostrum phase, including lactoransferrin and clusterin, which were both 2.8 fold lower at 7 d postpartum compared with the colostrum phase [5]. This was similar to our observation of higher abundances of immune-associate proteins in the MFGM proteome in M1 versus M14. Higher presence of several immune-associated proteins in M1, including not only immunoglobulins but also other proteins that play roles in pathogen detection and the immune response, were apparent through statistical analysis of protein abundances (Table 1) and GO comparison (Fig. 3). We further observed a lower abundance of vitamin D-binding protein, hemopexin, serum amyloid A, and lactadherin at M14 compared with M1. These 4 proteins are diverse in their actions and are ubiquitous in the body; however, they are all involved in supporting host immunity [44–46]. Conversely, butyrophilin subfamily 1 member 1A was higher in M14 compared with M1 in the current study. While butyrophilin subfamily 1 member 1A does belong to the immunoglobulin family [47], the higher abundance of this protein at M14 is not unexpected. As previously reported, this increase is likely due to its key role in milk fat globule synthesis in concert with perilipin 2 [38]. Both of these proteins were higher in M14 compared with M1.

**Fig. 4:**
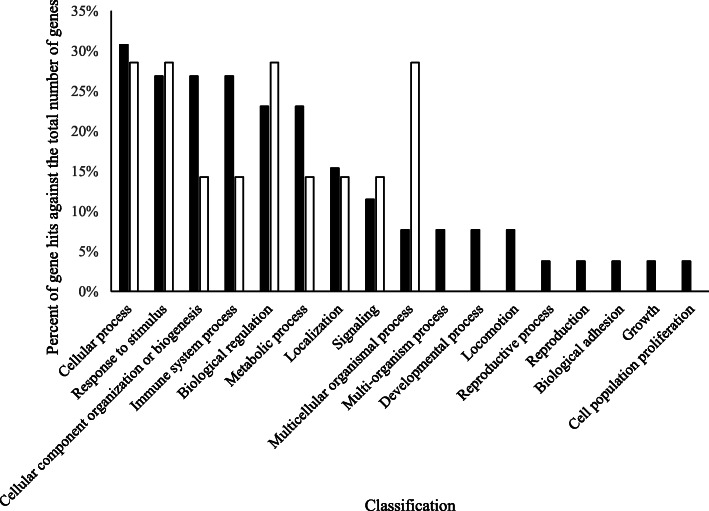
Biological processes of proteins that were impacted by parity and were higher in multiparous cows compared with primiparous cows (black bars), and lower in multiparous cows compared with primiparous cows (white bars)

Interestingly, butyrophilin subfamily 1 member 1A abundance may also be affected by PAR, as E1BHI7 was consistently higher in PP versus MP cows. However, peptide counts of another sequence linked with butyrophilin 1 member 1A (P18892) did not demonstrate this trend. This disparity may be a result of amino acid sequence overlap with other proteins and requires further research. Apart from this protein, PAR affected 46 proteins (Tables 3 and 4) in the current experiment. There is a paucity of published data characterizing the impact of PAR on the MFGM proteome. However, hypotheses surrounding the influence of PAR on the MFGM proteome can be formed based on our knowledge of other impacts of PAR on milk production, as well as known GO of identified proteins. MP cows are known to have higher milk and component yields than PP cows, likely leading to an overarching increase in the abundances of individual proteins associated with milk fat and protein synthesis and secretion in MP cows, many of which may be associated with the MFGM (factor 1 for consideration). Ultimately, the higher productivity of MP cows is normally due to higher rates of cell differentiation in the MP mammary gland compared to that of PP cows [48], which would suggest a higher abundance of secreted proteins related to mammary energetics and cellular metabolism in MP cows (factor 2 for consideration). In addition, the milk fat droplet size appears to be positively correlated to fat yield [49] and saturated fatty acid content [3, 50], which are higher in milk from MP cows [29, 51, 52]. It could therefore be extrapolated that MP cows may have increased secretion of proteins associated with mammary de novo fatty acid synthesis (factor 3 for consideration). Finally, the observation that MP cows typically secrete colostrum containing higher immunoglobulin content [29] may also elude to a more concerted drive to secrete immune-rich colostrum, including a more diverse array of immune-associated protein being secreted as part of the MFGM proteome (factor 4 for consideration). Overall, we observed that 84.8% of proteins affected by PAR were higher in milk from MP cows compared with PP cows, corroborating the suggestion that MP cows have higher secretory abundances of proteins compared with PP cows. Our additional observation of higher abundances of biogenic proteins (Table 3; Fig. 4), including histones 1H1E, H2A, H2B, H3, and H4, actin, macroglobulin, and vimentin in the MP cows compared with the PP cows supports the idea of chromatin remodeling and higher cellular differentiation [53–55].

The suggestion that proteins associated with de novo milk fat synthesis would be higher in MP cows did not appear to result in a higher secretory abundance of MFGM proteins associated with milk fat synthesis. While apolipoprotein E abundance was higher in samples from MP cows, additional protein differences were lacking. This was a somewhat surprising result, but perhaps speaks to the higher rate of intracellular metabolism in the PP gland compared with the MP gland, ultimately resulting in comparable presence of milk fat-associated proteins in the MFGM.

As discussed above, proteins involved in the immune system process were more represented in M1 compared with M14 (Fig. 3); however, they were also higher in samples from MP compared with PP cows (Fig. 4). Several immunoglobulin proteins, lactotransferrin, complement C3, vitamin D-binding protein, cathelicidin proteins (1 and 7), S100 and CD proteins (5, 14 and 177) were present at higher abundance in the MFGM proteome from MP compared with PP cows. This in line with our earlier postulation, and may need to be considered when selecting colostrum donor cows.

Three proteins affected by PAR (A6QNL0: monocyte differentiation antigen CD14; G3N0V2: keratin 1; F6QVC9: annexin 5) were not affected by MIL or MIL × PAR. While monocyte differentiation antigen CD14 and keratin 1 abundances were higher in milk from MP cows compared with PP cows, annexin 5 abundance was higher in milk from PP cows. Human keratin is a common contaminant in proteomic analyses; however, the presence of bovine keratin in milk is well documented [56]. Formation of a teat canal keratin plug occurs during the dry period, and is thought to be influenced by PAR [57]. It is feasible that the keratin concentration in milk is indeed also affected by PAR. The observation that annexin 5 was higher in milk from PP cows is a novel finding. Few published studies have investigated the relationship between annexin 5 and PAR; however, annexin 5 is reportedly important for maintenance of placental health and fetal survival in other species [58]. The assessment of milk annexin 5 concentration for use as a biomarker of reproductive health should be further investigated to validate its potential use.

The majority (73.5%) of proteins affected by the interaction between MIL and PAR demonstrated a pattern whereby the higher protein abundances secreted by MP cows was numerically apparent in M1 but gradually dissipated, resulting in similar protein abundances across MP and PP cows by M14. Lactotransferrin, S100 protein variants, vitamin D-binding protein, and immunoglobulin chain fractions and receptors all followed a similar pattern. The majority of these proteins are involved in immune activity. The implication of a higher presence of immune-associated proteins being affected by the interaction of MIL and PAR underscores the potential importance of selectivity of colostrum donors.

**Fig. 5:**
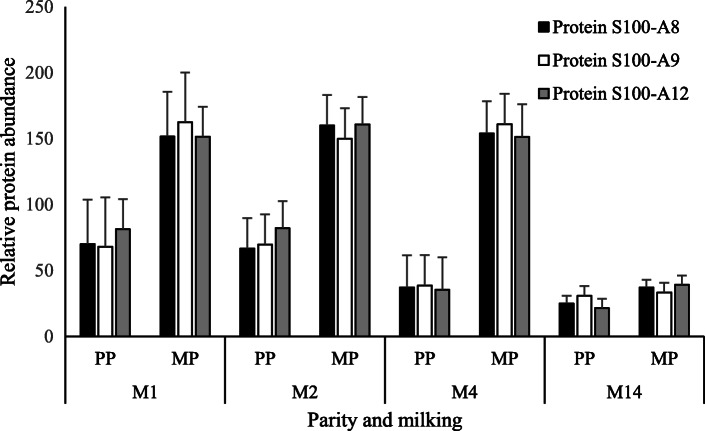
Abundances of S100 proteins affected by the interaction of milking and parity (MIL × PAR) from the MFGM identified by PANTHER in colostrum and milk samples collected from primiparous (PP, *n* = 10) and multiparous (MP, *n* = 10) cows at four milkings postpartum (M1, M2, M4, and M14)

It is interesting to note that S100 proteins are antimicrobial, and are also present in high abundance in the teat canal lining of dairy cattle [59]. Additionally, variants of S100 proteins serve as calcium sensors [60]. There is an increase in intracellular ionized calcium concentration at the onset of lactation [61]. This is also paired with the demand of calcium for milk production which further increases the need of calcium around parturition. Therefore, implications of calcium mobilization and demand may also contribute to the observed proteomic shifts. Protein S100 -A8, -A9, and -A12 were also affected by the interaction of MIL and PAR and their abundance patterns are presented in Fig. 5. Gelsolin, an abundant protein in the MFGM proteome, is also regulated by calcium [62] and followed a similar pattern to the S100 proteins. Comparative analysis of the bovine and human colostrum proteome using KEGG pathway characterization by Yang et al. [37] highlights the importance of calcium signaling pathways in bovine colostrum, and our results further contribute to our understanding of calcium-associated proteins in colostrum and factors that affect them.

There are a few exceptions of proteins that were affected by MIL × PAR and exhibited a higher abundance in colostrum of PP cows compared with MP cows, including apolipoprotein A-I, apolipoprotein C-III, serum amyloid A protein, and clusterin. The abundances of these proteins were similar across PP and MP cows by M14. Hérnandez-Castellano et al. [63] observed higher plasma abundances of apolipoprotein A-IV, B-100, and E in lambs fed colostrum 2 h after birth compared with lambs that were not fed colostrum until 14 h after birth. Given the importance of apolipoproteins in metabolic function [64], further research investigating the impact of feeding PP colostrum on the function of apolipoproteins in neonatal calves is warranted. Additionally, the observation of higher serum amyloid A abundance in the MFGM of MP cows as MIL increased is a novel finding. A review by Hérnandez-Castellano et al. [65] outlines the potential importance of serum amyloid A in colostrum due to its pro-inflammatory function. The current observation that serum amyloid A is affected by MIL × PAR may be important from a biomarker perspective to better monitor mammary pathogen loads.

## Conclusion

Seventy five percent of the MFGM proteome characterized in the current study was impacted by MIL, PAR, or their interaction, demonstrating the significant impact that these parameters have on the milk protein profile. Consistent with previous results, M1 samples were higher in immune-associated proteins and regulatory proteins. Contrary to our hypotheses, the MFGM from MP cows comprised higher abundances of proteins associated with cellular differentiation and immune function. In addition, proteins related to milk fatty acid synthesis or secretion were not different in abundance in the MFGM from MP cows compared with PP cows. The observed impact of MIL × PAR was also a novel and unexpected observation, and supports the need to further develop criteria to assess colostrum quality and parameters for selection of colostrum donors. These results also highlight the potential use of MFGM proteins for use as biomarkers of mammary function. Although results from the effect of MIL were not surprising, the impact of PAR, as well as the interaction of MIL and PAR, display a relationship that requires further investigation.

## Supplementary information

**Additional file 1.** Complete results dataset of LC-MS/MS from Proteome Discoverer 2.2.

## Data Availability

All data generated or analysed during this study are included in this published article and its supplementary information files.

## Abbreviations

CH_3_CN: Acetonitrile
FA: Formic acid
FDR: False-discovery rate
GO: Gene ontology
HCD: Higher-energy collisional dissociation
LC-MS/MS: Liquid chromatography-tandem mass spectrometry
M1: First milking postpartum
M2: Second milking postpartum
M4: Fourth milking postpartum
M14: Fourteenth milking postpartum
MFGM: The milk fat globule membrane
MIL: Milking
MIL × PAR: Milking × parity
MP: Multiparous
MS/MS: Tandem mass spectrometry
PAR: Parity
PBS: Phosphate buffered saline
PP: Primiparous
RIPA: Radioimmunoprecipitation assay
UC: Universal control
